# Management of patients with pulmonary mycobacteriosis in France: a multicenter retrospective cohort study

**DOI:** 10.1186/s12890-021-01701-5

**Published:** 2021-10-26

**Authors:** Pascale Bemer, Olivia Peuchant, Hélène Guet-Revillet, Julien Bador, Charlotte Balavoine, Damien Basille, Guillaume Beltramo, François-Xavier Blanc, Elodie Blanchard, Sarah Boulanger, Anne Bourgoin, David Boutoille, Emmanuelle Cambau, Frédérique Canis, Didier Caparros, Anne Carricajo, Christian Carrière, Gérard Couetdic, Francis Couturaud, Jean-Charles Dalphin, Tristan Degot, Marion Desquiens, Gilles Devouassoux, Jean-Marie Duez, Oana Dumitrescu, Magali Dupuy-Grasset, Alice Gaudart, Marjolaine Georges, Cendrine Godet, Sylvain Godreuil, Aurélie Guillouzouic, Farida Hamdad-Daoudi, Geneviève Héry-Arnaud, Christelle Koebel, Aurore Lagrange, Philippe Lanotte, Sylvain Marchand-Adam, Faïza Mougari, Marlène Murris, Isabelle Patry, Michèle Pérouse de Montclos, Laurent Raskine, Karine Risso, Christine Segonds, Dominique Sicard, Dominique Terru, Anne Vachée, Jean-Michel Vergnon, Christian Martin, Frédéric Schramm, Claire Andrejak

**Affiliations:** 1 Department of Bacteriology, CHU Nantes, Nantes, France; 2 Department of Bacteriology, CHU Bordeaux, Bordeaux, France; 3 Department of Bacteriology, CHU Toulouse, Toulouse, France; 4 Department of Bacteriology, CHU Dijon, Dijon, France; 5 Department of Respiratory Diseases, CHU Tours, Tours, France; 6 Department of Respiratory Diseases, Amiens University Hospital, CHU Amiens, 80054 Amiens Cedex 1, France; 7 EA 4294 AGIR, Picardie Jules Verne University, Amiens, France; 8 Department of Respiratory Diseases, CHU Dijon, Dijon, France; 9 Department of Respiratory Diseases, CHU Nantes, Nantes, France; 10 Department of Respiratory Diseases, CHU Bordeaux, Bordeaux, France; 11 Department of Respiratory Diseases, CH Roubaix, Bordeaux, France; 12 Department of Bacteriology, CHU Poitiers, Poitiers, France; 13 Department of Infectious Disease, CHU Nantes, Nantes, France; 14 Department of Bacteriology, AP-HP, Paris, France; 15 Laboratoire Associé du Centre National de Référence des Mycobactéries et Résistance des Mycobactéries aux Antibiotiques (CNR-MyRMA), Paris, France; 16 Department of Bacteriology, CH Valenciennes, Valenciennes, France; 17 Department of Respiratory Diseases, CH Valenciennes, Valenciennes, France; 18 Department of Bacteriology, CHU Saint Etienne, Saint Etienne, France; 19 Department of Bacteriology, CHU Montpellier, Montpellier, France; 20 Department of Bacteriology, CHU Besançon, Besançon, France; 21 Department of Respiratory Diseases, CHU Brest, Brest, France; 22 Department of Respiratory Diseases, CHU Besançon, Besançon, France; 23 Department of Respiratory Diseases, CHRU Strasbourg, Strasbourg, France; 24 Department of Respiratory Diseases, Hospices Civils Lyon, Lyon, France; 25 Department of Bacteriology, Hospices Civils Lyon, Lyon, France; 26 Department of Respiratory Diseases, CHU Limoges, Limoges, France; 27 Department of Bacteriology, CHU Nice, Nice, France; 28 Department of Respiratory Diseases, CHU Poitiers, Poitiers, France; 29 Department of Bacteriology, CHU Amiens, Amiens, France; 30 Department of Bacteriology, CHU Brest, Brest, France; 31 Department of Bacteriology, CHRU Strasbourg, Strasbourg, France; 32 Department of Bacteriology, CHU Tours, Tours, France; 33 Department of Respiratory Diseases, CHU Toulouse, Toulouse, France; 34 Department of Respiratory Diseases, CHU Nice, Nice, France; 35 Department of Bacteriology, CH Roubaix, Roubaix, France; 36 Department of Respiratory Diseases, CHU Saint Etienne, Saint Etienne, France; 37 Department of Bacteriology, CHU Limoges, Limoges, France

**Keywords:** Nontuberculous mycobacteria, Mycobacterium xenopi, Mycobacterium avium complex, Prognosis, Management

## Abstract

**Background:**

Recent studies report very low adherence of practitioners to ATS/IDSA recommendations for the treatment of nontuberculous mycobacteria pulmonary disease (NTM-PD), as well as a great variability of practices. Type of management could impact prognosis.

**Methods:**

To evaluate management and prognosis of patients with NTM-PD cases with respect to ATS recommendations, we conducted a multicenter retrospective cohort study (18 sentinel sites distributed throughout France), over a period of six years. We collected clinical, radiological, microbiological characteristics, management and outcome of the patients (especially death or not).

**Results:**

477 patients with NTM-PD were included. Respiratory comorbidities were found in 68% of cases, tuberculosis sequelae in 31.4% of patients, and immunosuppression in 16.8% of cases. The three most common NTM species were *Mycobacterium avium* complex (60%), *M. xenopi* (20%) and *M. kansasii* (5.7%). Smear-positive was found in one third of NTM-PD. Nodulobronchiectatic forms were observed in 54.3% of cases, and cavitary forms in 19.1% of patients*.* Sixty-three percent of patients were treated, 72.4% of patients with smear-positive samples, and 57.5% of patients with smear-negative samples. Treatment was in adequacy with ATS guidelines in 73.5%. The 2-year mortality was 14.4%. In the Cox regression, treatment (HR = 0.51), age (HR = 1.02), and *M. abscessus* (3.19) appeared as the 3 significant independent prognostic factors.

**Conclusion:**

These findings highlight the adequacy between French practices and the ATS/IDSA guidelines. Treatment was associated with a better survival.

**Supplementary Information:**

The online version contains supplementary material available at 10.1186/s12890-021-01701-5.

## Background

Nontuberculous mycobacteria pulmonary disease (NTM-PD) is not notifiable in France, as well as in most of the world. A large amount of epidemiological information remains unavailable. The microbiological diagnosis, even insufficient, is essential to make the diagnosis of infection. The clinical significance of a single positive sputum specimen in culture is difficult to estimate. It is important to follow the recommendations that at least two sputa or one bronchoalveolar lavage be culture-positive to consider microbiological criteria for NTM-PD [1, 2]. To do this, it is essential to correctly identify NTM at the species level [3]. The cost of management of NTM-PD has been evaluated four-fold higher than that for matched control, related to hospitalization for 63% and antibiotic treatment for 22% [4].

A comparison between different countries showed that nodulobronchiectatic forms were more frequent in Japan than in Europe (59.8% vs 39.2%, respectively), and that moderate to severe clinical forms were less common in Japan than in Europe (31% to 2% vs. 59% to 19%, respectively) [5]. Patients were more often treated in Europe than in Japan (68% vs 43%, respectively). In this study, it appears that in Europe, Italian and Spanish patients were more than six times as likely to be treated than patients in France. The most influential factors for treating NTM-PD were age > 60 years and severe clinical signs [5]. Two recent studies reported very low adherence of practitioners to ATS/IDSA treatment recommendations, particularly for *Mycobacterium avium* complex (MAC) and *M. abscessus* species, as well as a great variability of practices [5, 6]. But these studies have been performed with questionnaires fulfilled by physicians. It is difficult to know who are these physicians and if these physicians are representative of all physicians who take care of NTM-PD patients. The other point of view is to analyze management of NTM-PD patients and to compare to ATS/IDSA guidelines.

The purpose of this study was to evaluate management and prognosis of French patients with NTM-PD according to the 2007 ATS/IDSA recommendations over a period of six years, from January 2009 to December 2014. This study also described an overview of NTM-PD epidemiology in France.

## Methods

This study was designed as a multicenter, retrospective, observational, cross-sectional study of patients aged more than 18 years and with NTM-PD, conducted according to the STROBE guidelines. The study protocol was approved by institutional review board and ethics committee (PI2018_843_0049). We gave written information on the study to each patient. In case of opposition to participate, patient was not included. The potential opposition is indicated in the medical record.

The Mycobacteria Medical (MycoMed) Study Group is a French network of 18 microbiological laboratories. The main objectives of the MycoMed Study Group are i/ to promote exchanges on laboratory practices and diagnostic approach in Mycobacteriology as part of laboratory accreditation, ii/ to initiate clinical-biological studies. Two previous studies of this Group analyzed incidence of MAC pulmonary disease between 2000 and 2002 and incidence of NTM-PD in HIV-negative patients from 2001 to 2003 [7, 8].

Patients ≥ 18 years-old were included from January 2009 through December 2014 in 18 French Hospitals belonging to the MycoMed network.

All patients with at least one positive respiratory sample with NTM were eligible but only patients meeting the 2007 ATS/IDSA criteria after reviewing medical records were included. Patients with cystic fibrosis were excluded from the study. We have chosen 2007 ATS/IDSA criteria instead of 2017 BTS and 2020 ATS/IDSA/ERS/ESCMID, as our last was included in 2015, before publications of these 2 recent guidelines [2, 9].

Demographic, bacteriological and clinical and radiological data were obtained from medical records. For the primary survival analysis, the date of each patient’s first positive specimen was used to ascertain comorbidity history. This allowed us to avoid comorbidity ascertainment bias introduced by differential survival times.

Respiratory specimens were decontaminated using the NALC-NaOH method. Microscopic examination was performed by Zielh-Neelsen staining, and specimens were inoculated onto liquid culture medium (mainly BD BACTEC™ MGIT™ 960 system, Becton Dickinson) and a solid culture medium (mainly Löwenstein-Jensen). NTM identification at the species level was based on molecular characterization using commercial kits: AccuProbe® system (Gen-Probe, Inc., USA), INNO-LiPA Mycobacteria system (Innogenetics, Belgium), and GenoType Mycobacterium system (Hain Lifescience, Germany). Partial gene sequencing (*rpo*B/*hsp*65/16S-23S internal transcribed spacer, 16S rRNA) was less used. Since 2013, identification by matrix-assisted laser desorption/ionization time-of-flight mass spectrometry began to be carried out in some laboratories. In case of discrepancies or failures of identification, strains were sent to the French national reference center for mycobacteria.

We decided to group *M. intracellulare* and *M. chimaera* as some centers did not do at this time difference between the two species. For the same reason, we will present results for *M. abscessus* complex and not for each subspecies.

After descriptive analysis, univariate analysis was done for qualitative variables, Pearson’s Chi^2^ or Fisher exact test and for quantitative variables, Student t-test or Wilcoxon rank test. We ascertained mortality from the medical records. Follow-up time was computed from the index date that defined an NTM disease episode until the date of death, migration, last hospital consultation, 3 years after index date, or 1^st^ June 2017, whichever came first. We used Kaplan–Meier analysis with log-rank testing to calculate and compare crude cumulative survival according to study variables, and Cox regression (univariate and multivariate survival analysis). Statistical analyses were performed using SAS version 9.1 software.

## Results

During the period study, 568 NTM-PD were included. Among them 91 were excluded because of lack of radiologic data (87 cases) or identification of two different NTM species (4 cases). Overall, the remaining 477 cases (53.3% of males) met the 2007 ATS/IDSA criteria for NTM-PD, and were included. The median age was 65 years [range 18–96].

MAC comprised the majority (286/477, 60%) of all NTM findings (*M. avium*, n = 149; *M.intracellulare*, n = 134; MAC without subspecie identification, n = 3), followed by, *M.* *xenopi* (94/477, 19.7%), *M.kansasii* (27/477, 5.7%), *M.abscessus* complex (18/477,3.8%) *M.fortuitum* complex (17/477, 3.6%) (*M.* *fortuitum* (n = 13; *M. chelonae*, n = 4). Other unfrequent NTM species were identified in 5.4% (26/477) of cases: *M. simiae* (n = 10), *M. malmoense* (n = 4)*, M.* *genavense*, *M.* *scrofulaceum* and *M.* *szulgai* in two cases for each species while *M. arupense*, *M. florentinum M.* *heckeshornense, M. interjectum, M. lentiflavum* and *M. shimoidei* were only isolated once. In the remaining 9 cases, NTM could not be identified at the species level.

### Clinical characteristics

MAC patients were a majority of female (56.2%) whereas *M. xenopi* and *M. kansasii* patients were a majority of males (60.9% and 70.4%, respectively). Sixty-eight percent of patients had respiratory comorbidities (chronic obstructive pulmonary disease, bronchiectasis) and 31.4% had tuberculosis sequelae. Few patients (16.8%) had immunosuppression, mainly because of essentially systemic corticosteroids, immunosuppressive drugs or cancer. Forty-one patients (8.6%) were HIV-positive and only 7.7% of them had immunosuppression (less than 200 CD4/mm^3^).

### Radiographical findings

More than half of patients (259/477, 54.3%) presented nodulobronchiectatic pattern (Table 1). Cavities were reported on 19.1% of cases while other radiological findings (such as pleural effusion or interstitial lesions) were found in 26.6% of NTM-PD. For this 26.6%, lesions are not the classically pattern described in ATS/IDSA 2007. We considered them as NTM-PD, as all these patients underwent a complete evaluation, and the only possible diagnosis was NTM-PD despite the atypical radiological presentation. Nevertheless, a misclassification bias is always possible. This limit reflects one usual problem in real life.

**Table 1:** Radiological findings according to the NTM species

	Smear-positive Nb (%)	Cavitary forms Nb (%)	Nodulo-bronchiectatic Forms Nb (%)	Other findings Nb (%)
*Mycobacterium avium* complex (n = 286)	98 (34.3)	46 (16.1)	169 (59.1)	71 (24.8)
*Mycobacterium xenopi* (n = 94)	39 (41.5)	32 (34.0)	40 (42.6)	22 (23.4)
*Mycobacterium kansasii* (n = 27)	11 (40.7)	7 (25.9)	15 (55.6)	5 (18.5)
*Mycobacterium abscessus* complex (n = 18)	11 (61.1)	2 (11.1)	10 (55.6)	6 (33.3)
*Mycobacterium fortuitum* complex (n = 17)	3 (17.6)	0 (0.0)	8 (47.1)	9 (52.9)
Other species (n = 35)	13 (37.1)	4 (11.4)	17 (48.6)	14 (40.0)
Total (n = 477)	175 (36.7)	91 (19.1)	259 (54.3)	127 (26.6)

The different NTM had significant difference in term of radiological pattern (*p* = 0.001; Table 1). Nodulobronchiectatic pattern was the predominant radiological pattern in subjects infected by MAC (59.1%), *M.* *kansasii* (55.6%) and *M. abscessus* complex (55.6%). A slightly higher rate of nodulobronchiectatic pattern (42.6%) than cavitations (34%) was observed in patients with NTM-PD cause by *M. xenopi*. In the 17 NTM-PD du to *M.* *fortuitum* complex, other forms were reported in 52.9% of cases and nodulobronchiectatic in 47.1% of cases.

### Microbiological results

#### Bacteriological load among NTM species

Smear-positive were found in 36.7% of the culture-positive specimens Interestingly, results depended on NTM species: 61.1% of specimens with *M.* *abscessus* complex were smear-positive, 41.5% for *M. xenopi,* 40.7% for *M. kansasii,* 34.3% for MAC and 17.6% for *M.* *fortuitum* complex.

#### Samples recovered

The median number of positive sample was two with variations from 1 to 11 positive specimens. For the 132 patients with only one positive sample, the specimen was always a bronchoalveolar lavage. When compared with patients with at least two positive samples, only 28 patients had a smear-positive specimen in the patients with unique positive BAL subgroup (21.2% vs. 42.6% (n = 147/345), *p* < 0.0001). These patients, with a unique positive BAL, had significantly less often a cavitary form (7.6% versus 23.5%, *p* < 0.0001) and have been treated less frequently (48.5% vs. 68.4%).

#### Treatment (Table 2)

**Table 2:** Distribution of treated patients according to the species and the results of microscopy

	Treated patients		
	Total available data	Positive-smear results N/A* (%)	Negative-smear results N/A* (%)
*Mycobacterium avium* complex	280	73/96 (76.0)	115/184 (62.5)
*Mycobacterium xenopi*	53	24/36 (66.7)	29/55 (52.7)
*Mycobacterium kansasii*	18	8/11 (72.7)	10/13 (76.9)
*Mycobacterium fortuitum* complex	4	2/3 (66.7)	2/14 (14.3)
*Mycobacterium abscessus* complex	13	8/11 (72.7)	5/7 (71.4)
Other species	16	8/13 (61.5)	8/21 (38.1)
Total	292	123/170 (72.4)	169/294 (57.5)

N/A*: number of results among available data

Treatment data were available for 464 patients (Table 2). Overall, 62.9% (292/464) of patients were treated. Patients with smear-positive specimens received more often treatment than those smear-negative specimens (72.4% vs 57.5%, respectively *p* = 0.001). About two-third of subjects with MAC NTM-PD were treated, ranging from 76.0% in smear-positive cases to 62.5% in smear-negative cases (*p* = 0.02). Patients infected with *M. xenopi* were treated in 66.7% of smear-positive and 52.7% of smear-negative cases. Three-quarters of patients with *M. kansasii* or *M. abscessus* complex lung disease were treated regardless of smear-positivity.

Patients with NTM-PD due to *M. fortuitum* complex were treated in 23.5% of cases, mostly when specimens were smear-positive. These result were concordant with the last ERS/ATS/IDSA/ESCMID guidelines where it is suggested that initiation of treatment is preferred rather than watchful waiting, in the context of positive acid-fast bacilli sputum smears [2].

Treatment was in adequacy with 2007 ATS/IDSA guidelines in 73.5% of cases [1]. Eighty-nine percent of patients were treated with at least 3 drugs for more than 12 months in 75.1% of cases). Clarithromycin was used in 90.5% of MAC infections, 76.5% of *M. xenopi* and 84.6% of *M. abscessus* complex infections. Amikacin was mostly used in *M. abscessus* complex infections (58.3% vs. 11.6% in MAC group and 8.3% in *M. xenopi*). Clarithromycin appeared as the most used macrolides in France, probably because it is the only one with an official indication for NTM treatment. Rifampicin and ethambutol were used in a vast majority of NTM-PD cases except those caused by *M. abscessus* complex**.**

#### Outcome

**Fig. 1:**
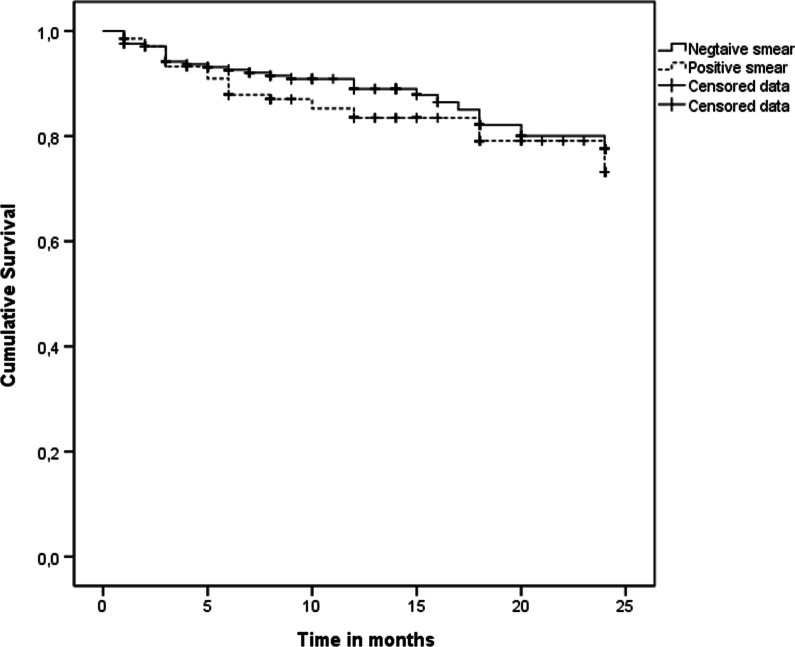
Cumulative survival according to smear results

For the 418 patients with available data, the 2-year mortality was 14.4%. In survival univariate analysis, smear result did not constitute a pejorative prognostic factor (HR 1.33 [0.78–2.26], Table 3, Fig. 1). Radiological form seemed not to be a prognostic factor. Treatment, age, and *M. abscessus* appeared as the 3 significant independent prognostic factors (Table 3) in the Cox regression.

**Table 3:** Survival analysis, according to Cox regression

	Non adjusted HR [CI 95%]	*p*	Adjusted HR [CI 95%]	*p*
Age	1.02 [1.01–1.04]	0.02	1.02 [1.01–1.04]	0.04
Female	0.73 [0.42–1.27]	0.27		
Respiratory diseases	0.99 [0.54–1.83]	0.99		
Past mycobacterial diseases	1.56 [0.89–2.71]	0.11		
Cavitary disease	0.84 [0.42–1.66]	0.61		
Positive smear	1.33 [0.78–2.26]	0.30		
NTM specie				
MAC	Reference		Reference	
*M. xenopi*	1.33 [0.68–2.60]	0.40	1.21 [0.59–2.49]	0.61
*M. abscessus*	2.64 [1.03–6.78]	0.04	3.19 [1.22–8.32]	0.02
*M. kansasii*	0.69 [0.16–2.88]	0.61	1.12 [0.20–3.45]	0.83
Others	1.16 [0.41–3.29]	0.78	1.02 [1.01–1.04]	0.83
Treatment	0.47 [0.27–0 .83]	0.009	C [0.28–0.92]	0.03

## Discussion

Previous studies on NTM-PD are often general reviews mainly on the MAC complex [3, 10], or epidemiological studies based on laboratory data without clinical or radiological criteria [11, 12]. Few cohort studies met ATS criteria [13, 14].

Our cohort included 477 patients who met the 2007 ATS/IDSA criteria for NTM-PD from 18 centers between 2009 and 2014. Only two previous French studies reported data on 101 pulmonary MAC infections from 2000 to 2002, and on 262 NTM-PD from 2001 to 2003 according to the 1997 ATS criteria [7, 8]. Our study is one of the biggest cohort studies of NTM diseased patients and allows us to have real conclusions about NTM-PD epidemiology and management in France.

Unfortunately, this study did not include all NTM-PD cases in France as only 18 centers participated, and does not permit to calculate the exact incidence and prevalence in France. Nevertheless, these 18 centers from the MycoMed network are among the biggest hospitals in France. One other limitation was the delay between the last included patients and the final analysis. Indeed, as it is retrospective study, we needed some administrative authorizations, and some time to give information to all concerned patients.

The diagnostic practices are homogeneous and standardized in these 18 centers, responding to published recommendations [15, 16], and identical to European practices [11]. Solid and liquid culture media were used as recommended [15]. The majority of NTMs were identified at the species level, most often using line probe hybridization, more rarely on-demand sequencing, or recently mass spectrometry [15, 16].

The following quality criteria can be highlighted for our cohort: i/ less than 2% of the strains could not be identified to the species level, ii/ it does not contain any contaminating species such as *M. gordonae* or *M. terrae* complex [1], iii/ finally very few pulmonary *M. fortuitum* complex infections, most often considered as non-pathogenic, were included [17].

In our cohort, the species distribution of NTM was close to that reported earlier in France [8] and more recently in a survey study of NTM in the European Union, which included France [18]. MAC was the commonest group of cultured organisms, as reported worldwide [18]. While studies showed that *M.* *avium* was the predominated subspecies recovered from human specimens [7, 18] in Europe, we found a similar number of *M.* *avium* and *M.* *intracellulare* isolates in our cohort. *M.* *xenopi,* followed by *M. kansasii,* were the second and the third species, respectively, the most frequently encountered in France. In the collaborative study of the NTM-NET network, *M. abscessus* complex and M. *kansasii* were the second and the third species, respectively.

The analysis of radiological and microbiological aspects of pulmonary NTM highlighted the variability associated with the species, and with the study period. Regarding MAC, the two-series meeting the ATS 1990 criteria of Obayashi and Wright reported a percentage of direct-smear positive patients greater than 50%, especially in the case of upper lobe cavitary lesions [13, 19]. In the cohort of Wright [17], microscopy examination of pulmonary NTM due to MAC, *M. abscessus* and M*. kansasii* was positive in 58%, 51% and 78% respectively. By comparison, in two subsequent studies of MAC meeting the 2007 ATS criteria, the percentages of cavitary lesions and positive direct examinations were 18% and 31% in the study of Boyle [12], and 20% and 35% in the study of Ito [20]. Our cohort was characterized by 19.1% of cavitary lesions and 36.7% of smear-positive results, similar to previous studies [14, 20].

The decrease of these two criteria is probably due to the lack of positive direct examination in the 2007 ATS criteria.

In our cohort, we found a high (61%) percentage of smear-positive samples and a small (11%) percentage of cavitary lesions in NTM-PD due to the *M.* *abscessus* complex. In comparison, the Wright study also reported a high percentage of positive direct examinations of 51.3% in NTM due to *M. abscessus* [19]. Specific studies were conducted on *M. kansasii* lung infections. In the Wright series, 29 (78.4%) of 37 specimens positive in culture for *M. kansasii* were smear positive. The 37 specimens concerned only 8 patients for whom the radiological data were not known [19]. *M. kansasii* is an NTM so far known to present a similar clinical picture to that of pulmonary tuberculosis with fibrocavitary lesions in the upper lobes [1]. A recent study reviewing clinical manifestations and radiographic findings of patients with *M. kansasii* respiratory isolates provided further clarification [21]. Indeed, among 54 patients who met the ATS/IDSA diagnostic criteria, 44% exhibited the fibrocavitary form and 32% the nodulo-bronchiectatic form [21]. Our series even showed that up to 55% of *M. kansasii* NTM-PD developed nodular bronchiectatic forms. It is very likely that the change in ATS criteria in 2007 has led to the diagnosis of more nodular forms among *M. kansasii* NTM-PD.

Nearly half of *M. xenopi* were direct-smear positive for 34% cavitary forms. These results were closely related to those published in 2009 [22], reporting 61% of positive direct examinations and 31% of cavitary forms among the 136 M*. xenopi* pulmonary NTM in France.

Finally, the least NTM, with some positive smearin our series, were due to *M. fortuitum* (17.6%). In the Park study, direct examination was positive in 9/26 patients (35%) with at least three positive cultures to *M. fortuitum* [17].

Overall, 62.9% of patients were treated in our cohort, with 72.4% smear-positive specimens. The prevalence of treated patients depends on the studies. In two cohorts of MAC NTM-PD, 46.1% and 53.1% of patients received antimicrobial therapy respectively *vs* 67.1% in our study of which 62.5% were smear-negative [14, 20]. Seventy-five and 72.2% of *M. kansasii* and *M. abscessus* complex infections were treated respectively, regardless of whether the patients were smear-positive or not, which correlates well with the recognized pathogenicity of these species [1, 2, 23]. For *M. xenopi*, 54.6% of patients received treatment, which is more than the 32% reported in a previous French study [22]. But in the French study, one third of patients were very immunosuppressed (AIDS with less than 50 CD4/mm^3^) and a vast majority of them died before bacteriological diagnosis. Finally, only 4 (23.5%) patients infected with *M. fortuitum* were treated. Results obtained by Park et al. showed that there was no clinical worsening or persistent positive cultures among the 25 untreated patients on a 12-month follow-up [17]. These data confirm the low pathogenicity of this species, most often responsible of colonization or transient infection that does not require generally a treatment [1, 23].

Our results highlight the conformity of practices of French clinicians with the ATS/IDSA guidelines. Most patients received at least three drugs during 12 months or more. These results are in contrast with those of US and European countries [5, 6]. These differences could be partly explained by the study methodology and physician expertise. These physicians were randomly surveyed to generate nationally representative analyses of practice. These physicians included practitioners outside of reference centers and in non-specialized fields. These adequacy between French practices and ATS/IDSA guidelines found in our study could maybe explain the good prognosis of treated patients in our cohort in contrast to literature data especially for MAC and *M. xenopi* [22, 24, 25]. Indeed, a more favorable evolution was observed in our treated patients, whether or not patients are positive for direct examination. The decision to initiate a treatment should be individualized, based on clinical factors, radiological lesions, and some microbiological data (NTM specie, number and type of positive samples, and probably positive smear, often associated with cavitary lesions), as recommended by the 2 recent NTM guidelines [2, 9]. In our study, the treatment rate was high, but we have strictly selected patients: all have ATS/IDSA NTM disease diagnosis criteria, with a low rate of patients with an unique smear negative BAL. Moreover, all patients were clinically and radiologically symptomatic. The risk of treatment nonadherence is higher in patients with few symptoms, that is why treatment decision should be discussed according to patient and disease characteristics and with patient point of view. We have chosen to classify in more or less than 12 months, even if recommendations is 12 months after sputum conversion, because it is not easily, in the real life to have the date of sputum conversion..Finally, our lower mortality in comparison to previous published studies could be too due to patients characteristics or management differences: diagnosis or treatment prior to ATS/IDSA 2007 publication, less common use of macrolides [22]), predominance of observation without treatment [25], type of comorbidities (for example systemic immunosuppression [22] or NTM species included (especially *M. xenopi* [22, 25]. Indeed, in some others studies [26, 27], outcome was mainly associated with age, comorbidities and not with species (except *M. xenopi* associated with a poor prognosis) [26]. In our study, *M. abscessus* appeared with the poorest prognosis. Nevertheless, it is difficult to conclude, as only few patients with *M. abscessus* have been included in this cohort (n = 12) and no differentiation between *M. abscessus* stricto sensu and *M. massiliense* is available.

## Conclusion

This study based on collaboration between microbiologists and clinicians gives us very complete clinical, radiological, microbiological, treatment and outcome data. The three NTM species involved in pulmonary disease in France between 2009 and 2014 were MAC, *M. xenopi* and *M. kansasii*. This cohort study highlighted the role of adherence to treatment guidelines in prognosis, with an individual discussion of treatment or watchful waiting. In case of treatment, this treatment for slow growing NTM, as actually recommended, should be included at least 3 drugs for a duration of 12 months after sputum conversion. The main limitation will be the realization of control samples.

## Supplementary Information

**Additional file 1**. Guide to fulfill CRF.

## Data Availability

The datasets used and/or analysed during the current study are available from the corresponding author, Claire ANDREJAK (andrejak.claire@chu-amiens.fr)on reasonable request.

## Abbreviations

AIDS: Acquired Immunodeficiency Syndrom
ATS/IDSA: American Thoracic Society / Infectious Diseases Society of America
BAL: Bronchoalveolar Lavage
BTS: British Thoracic Society
HIV: Human Immunodeficiency Virus
MAC: *Mycobacterium* *avium* Complex
NTM: Nontuberculous mycobacteria
NTM-PD: Nontuberculous mycobacteria pulmonary disease
