# Supplementary material for: Management of patients with pulmonary mycobacteriosis in France: a multicenter retrospective cohort study
Source: BMC Pulm Med. 2021 Oct 26;21:333. doi: 10.1186/s12890-021-01701-5 (PMC8549171; doi:10.1186/s12890-021-01701-5)
Supplement: Supplementary file 1 — Additional file 1. Guide to fulfill CRF. [file 12890_2021_1701_MOESM1_ESM.docx]

**Guide to fulfill the CRF**

**N° (number of center-number of patients-initials)**

**Date of birth (month/year)**

**Date of diagnosis (month/year)**

**Sex**

**Inclusion criteria:**

- NTM pulmonary infection according to 2007 ATS/IDSA criteria
- Older than 18 years old

**Absence of exclusion criteria: no cystic fibrosis and opposition to participate**

**Comorbidities:**

- Previous mycobacterial disease
- Respiratory comorbidities (COPD, bronchiectasis, sarcoidosis)
- General immunosuppression
  - HIV infection
  - Cancer
  - Transplantation
  - Immunosuppressive drugs
  - Systemic corticosteroids
- HIV infection, if yes: CD4 count :

**Diagnosis:**

- Presence of clinical signs (cough, sputum, dyspnea, hemoptysis, fever, weight loss, fatigue)
- Radiological lesions:
  - Nodulobronchiectatic lesions
  - Cavities
  - Pleural effusion
  - Interstitial lesions
  - Others:
- Microbiological data:
  - Number of positive sample
  - For each sample: type and smear results
  - Specie:
    - MAC, with precision of the specie in the complex if known
      - *M. avium*
      - *M. intracellulare*
    - *M. abscessus* with precision of the specie in the complex if known
    - *M. xenopi*
    - *M. kansasii*
    - *M. fortuitum* with precision of the specie in the complex if known
    - M*. genavensae*
    - *M. simiae*
    - *M. malmoense*
    - *M. szulgai*
    - *M. scrofulaceum*
    - *M. gordone*
    - Other: precise which :
  - Antibiotic susceptibility available ? If yes: precise susceptibility and MIC of each tested drug if known

**Management:**

- No treatment
- Medical treatment; if yes:
  - Which drugs (for each duration of use)
    - Rifampicin
    - Rifabutin
    - Ethambutol
    - Clarithromycin
    - Azithromycin
    - Aminoglycosids
    - Fluoroquinolones: moxifloxacin, levofloxacin, ofloxacin, ciprofloxacin
    - Beta-lactams : imipenem, cefoxitine, others
    - Trimetroprin-sulfametoxazole
    - Linezolid
    - Clofazimin
    - Others drugs:
- Surgical treatment
  - If yes : which

**Outcome**

- Date of death, if death occurred
- Date of the last news
- Cure
- Relapse
- Failure
- Sequelae
